# The *Aspergillus flavus* Histone Acetyltransferase AflGcnE Regulates Morphogenesis, Aflatoxin Biosynthesis, and Pathogenicity

**DOI:** 10.3389/fmicb.2016.01324

**Published:** 2016-08-30

**Authors:** Huahui Lan, Ruilin Sun, Kun Fan, Kunlong Yang, Feng Zhang, Xin Y. Nie, Xiunai Wang, Zhenhong Zhuang, Shihua Wang

**Affiliations:** Key Laboratory of Pathogenic Fungi and Mycotoxins of Fujian Province, The Ministry of Education Key Laboratory of Biopesticide and Chemical Biology, and School of Life Sciences, Fujian Agriculture and Forestry UniversityFuzhou, China

**Keywords:** *A. flavus*, histone acetyltransferase, *AflgcnE*, aflatoxin, pathogenicity

## Abstract

Histone acetyltransferases (HATs) help regulate fungal development and the production of secondary metabolites. In this study, we determined that the HAT AflGcnE influenced morphogenesis and aflatoxin biosynthesis in *Aspergillus flavus.* We observed that AflGcnE localized to the nucleus and cytoplasm during the conidial production and germination stages, while it was located mainly in the nucleus during the hyphal development stage. Deletion of *AflgcnE* inhibited the growth of *A. flavus* and decreased the hydrophobicity of the cell surface. The Δ*AflgcnE* mutant exhibited a lack of asexual sporulation and was unable to generate sclerotia. Additionally, *AflgcnE* was required to maintain cell wall integrity and genotoxic stress responses. Importantly, the Δ*AflgcnE* mutant did not produce aflatoxins, which was consistent with a significant down-regulation of aflatoxin gene expression levels. Furthermore, our data revealed that *AflgcnE* is a pathogenicity factor required for colonizing maize seeds. In summary, we revealed that *A. flavus* AflGcnE is crucial for morphological development, aflatoxin biosynthesis, stress responses, and pathogenicity. Our findings help clarify the functional divergence of GcnE orthologs, and may provide a possible target for controlling *A. flavus* infections of agriculturally important crops.

## Introduction

The regulation of genes in eukaryotic cells involves dynamic chromatin rearrangements. For example, heterochromatin is involved in silencing and activating gene expression (Fischle et al., 2003). The effects of heterochromatin on gene expression are primarily achieved through histone post-translational modifications, including phosphorylation, methylation, ubiquitination, SUMOylation, and acetylation. Acetylation was the first chromatin modification process to be identified, and it has since become one of the most thoroughly studied epigenetic processes (Heintzman et al., 2009). Histone acetylation strongly affects nuclear activities such as DNA replication, DNA repair, and gene transcription. It also regulates several cellular processes in animals, plants, and fungi, including survival, cell proliferation, differentiation, and motility (Struhl, 1998; Brosch et al., 2008; Choudhary et al., 2014). Histone acetylation generally has two roles during the regulation of gene transcription. It alters the physical properties of DNA–histone interactions and provides a frame that binds to proteins and remodels chromatin (Spedale et al., 2012). Histone acetylation is a reversible process, and is controlled by histone acetyltransferases (HATs) and histone deacetylases. Histone hyperacetylation often favors the formation of euchromatin and leads to gene activation, while hypoacetylation favors heterochromatin formation and leads to gene inactivation. Therefore, characterizing the enzyme functions that regulate acetylation is an important way to clarify the critical roles of this epigenetic process.

The Spt-Ada-Gcn5-acetyltransferase (SAGA) complex is involved in the transcriptional regulation of 12% of the yeast genome (Lee et al., 2000). The Gcn5 core subunit is responsible for the acetyltransferase activity of the SAGA complex (Grant et al., 1997). Homologs of *Saccharomyces cerevisiae* Gcn5 have been observed to regulate growth, development, stress resistance, genome integrity, and host invasion in various fungal species. For example, *Candida albicans* Gcn5 is required for morphogenetic and stress responses (Chang et al., 2015). Additionally, deletion of *Ustilago maydis gcn5*, which influences dimorphism and virulence, results in long mycelial cells and fuzz-like colonies (González-Prieto et al., 2014). The *Aspergillus nidulans gcnE* null mutant produces abnormal conidiophores (Cánovas et al., 2014). *gcnE* is required for inducing the expression of the orsellinic acid gene cluster in bacteria (Nützmann et al., 2011). In *Cryptococcus neoformans*, Gcn5 is an important HAT that regulates the expression of specific genes such as *Kre61*. This gene encodes a β-glucan synthase involved in cell wall biosynthesis, which enables the fungus to respond appropriately to human hosts (O’Meara et al., 2010). Despite such extensive analysis of the roles of Gcn5 homologs, the function of GcnE in *Aspergillus flavus* has not been fully characterized.

*Aspergillus flavus* is an opportunistic fungal pathogen of oil crops, and is known for its production of aflatoxins in maize, peanuts, and tree nuts pre- and post-harvest (Amaike and Keller, 2011). Additionally, this fungus is also an opportunistic animal pathogen that causes aflatoxicosis and liver cancer (Hedayati et al., 2007). As a serious contaminant for crop production and animal husbandry, *A. flavus* is responsible for the loss of billions of dollars worldwide (Klich, 2007). Although the aflatoxin gene cluster has been extensively characterized, how aflatoxin biosynthesis is regulated has not been fully determined. Studies have revealed that the induction of aflatoxin biosynthesis is a complicated process (Bhatnagar et al., 2003; Georgianna and Payne, 2009; Amaike and Keller, 2011; Amare and Keller, 2014) that requires several transcription factors and signaling proteins (Duran et al., 2007; Chang et al., 2011; Amaike et al., 2013; Chang and Ehrlich, 2013). Additionally, chromatin regulation is involved. For example, the acetylation of histone H4 mediates aflatoxin biosynthesis in *Aspergillus parasiticus* (Roze et al., 2007), while *A. nidulans* regulates sterigmatocystin production through the acetylation of H3K9 (Nützmann et al., 2011) and H4K12 (Soukup et al., 2012). Recently, we described the potential roles of a DNA methyltransferase in aflatoxin production (Yang et al., 2016). However, whether HATs regulate aflatoxin biosynthesis remains unclear.

In this context, we investigated the functions of *A. flavus* Gcn5 orthologs regarding morphogenesis, aflatoxin biosynthesis, stress resistance, and seed colonization. We identified an indispensable role for AflGcnE in the regulation of developmental processes affecting growth rate, sporulation, sclerotial formation, stress resistance, seed colonization, and aflatoxin biosynthesis. AflGcnE may be a candidate target for preventing *A. flavus* infections of agriculturally important crops.

## Materials and Methods

### Strains and Culture Conditions

Fungal strains and plasmids used in this study are listed in **Supplementary Table S1**. Strains were grown at 37°C for growth test, and cultured at 28°C for aflatoxin analysis (aflatoxin-inducing temperature, as described; Zhang et al., 2015). All plates and flasks were cultured in dark condition. Each strain was grown on five plates or flasks, and each experiment was repeated three times.

### Sequence Resource and Phylogenetic Tree Analysis

Amino acid sequences of Gcn5 protein (*Aspergillus* spp. and *S. cerevisiae*, *C. albicans*, *Fusarium graminearum*, *Magnaporthe oryzae*, *Arabidopsis thaliana*, *Drosophila melanogaster*, *Mus musculus*, *Homo sapiens*) were downloaded from National Center for Biotechnology Information resources (NCBI^1^). The visualized Gcn5 domain was constructed by DOG 2.0 software (Ren et al., 2009). These Gcn5 protein sequences were aligned by ClustalW method, using MEGA 5.0 software, and a neighbor-joining phylogenetic tree was constructed. The GenBank accession numbers of organisms are presented in phylogenetic tree.

### Disruption of *AflgcnE* and Construction of the *AflgcnE-C* Complemented Strain

*Aspergillus flavus* gene deletion and transformation experiments were conducted using previously described protocols (Chang et al., 2010). The primers used for the *AflgcnE* gene knockout are listed in **Supplementary Table S2**. An overlap polymerase chain reaction (PCR) method was used to construct *AflgcnE* homologous fragments as previously described (Szewczyk et al., 2006). A 1,318-bp fragment upstream of *gcnE* was amplified with primers *gcnE*/AF and *gcnE*/AR, and a 1,313-bp fragment downstream of *gcnE* was amplified using primers *gcnE*/BF and *gcnE*/BR. A *pyrG* selection marker was amplified from *Aspergillus fumigatus* genomic DNA with primers *pyrG*/F and *pyrG*/R. A fusion PCR program and nested primers (*gcnE*/NF and *gcnE*/NR) were used to generate a fragment containing the up- and downstream segments and the *pyrG* selection marker. The fusion PCR products were purified and incorporated into PTSΔ*ku70*Δ*pyrG* strain protoplasts using an established procedure (Zhuang et al., 2016). To generate a Δ*AflgcnE-C* complemented strain, a commercial *Aspergillus* chromosome-integrating shuttle pPTRI vector (Takara, Japan) was digested with *Kpn*I (Thermo Fisher Scientific, USA). A 3.5-kb PCR product (1.5-kb *gcnE* coding sequence and 2.0-kb upstream sequence) was amplified from *A. flavus* CA14 genomic DNA using primers *gcnE*/CF and *gcnE*/CR, which contain the *Kpn*I recognition site. The digested pPTRI vector and PCR products were recombined using T4 DNA ligase (Takara). The accuracy of the recombinant pPTR-*gcnE* vector sequences was verified by DNA sequencing. The confirmed pPTR-*gcnE* vectors were then used to transform Δ*gcnE* protoplasts. All fungal transformants were analyzed by PCR and reverse transcription PCR (RT-PCR).

### Mycelial Growth and Analyses of Conidia and Sclerotia

Mycelial plugs (5 mm diameter) for each strain were removed from the periphery of a 2-day-old colony growing on yeast extract–sucrose (YES) solid medium. The plugs were used to inoculate potato dextrose agar (PDA; Becton Dickinson, France), YES agar (Yang et al., 2016), Czapek agar (CA; Becton Dickinson), and glucose minimal medium (GMM) agar (Shimizu and Keller, 2001). Cultures were incubated at 37°C in darkness, and colony diameters were measured daily. After 5 days, conidia were collected in triplicate from a 10-mm fungal plug removed from equivalent zones of the PDA and GMM agar media. The collected samples were homogenized and diluted in 3 ml 0.05% Tween-20. Conidia were counted using a hemocytometer and microscope. To analyze sclerotia, each strain was grown in Wickerham (WKM) agar medium (Raper and Thom, 1949) at 37°C in darkness. After 7 days, each plate was sprayed with 75% ethanol to wash away mycelial mats. Sclerotia were collected, frozen in liquid nitrogen, and lyophilized to measure the dry weight (Affeldt et al., 2014). Five plates were analyzed for each strain, and each experiment was repeated three times.

### Stress Assay

Mycelial plugs were used to inoculate YES agar medium supplemented with the following agents: cell wall stress agents calcofluor white (CFW, 200 μg/ml) and Congo red (CR, 1 mg/ml), hyperosmotic stress mediators sodium chloride (NaCl, 1 M) and potassium chloride (KCl, 1 M), genotoxic agents hydroxyurea (HU, 10 mM) and methyl methanesulfonate (MMS, 0.01%, v/v), and oxidative stress agents hydrogen peroxide (H_2_O_2_, 15 mM) and *tert*-butyl hydroperoxide (tBOOH, 0.5 mM). Inoculated plates were incubated at 37°C in darkness for 5 days. Colony diameters were measured every day. Each strain was cultured on five plates, and each experiment was repeated three times.

### Determination of Aflatoxin B Production

Aflatoxin B (AFB) production was measured by thin-layer chromatography (TLC) and high performance liquid chromatography (HPLC) analysis as previously described (Yang et al., 2016). Briefly, mycelial plugs (5 mm diameter) of the wild-type (WT), Δ*AflgcnE*, and Δ*AflgcnE-C* strains were used to inoculate 50 ml YES liquid medium, and cultures were incubated at 28°C. After 72 h, the cultures were combined with 25 ml chloroform in 250-ml flasks, which were shaken for 30 min. The mycelia were then collected, dried completely, and weighed. Next, the organic layer of each sample was added to a new plate, completely dried, and resuspended in chloroform solvent (1 μl/mg of mycelia). The extracts (10 μl/sample) were loaded onto silica TLC plates (Haiyang Chemical, Qingdao, China) and separated in developing solvent (chloroform:acetone = 9:1). The TLC plates were exposed to UV radiation (365 nm) and photographed using a Quantum ST5 imaging system (Vilber Lourmat Deutschland GmbH, Eberhardzell, Germany).

For HPLC analysis, the aflatoxin extracts were dissolved in methanol, filtered (0.22 μm), and analyzed using a Mycotox^TM^ column (Waters, Milford, MA, USA) at 42°C. The column was equilibrated in running solvent (56:22:22, water:methanol:acetonitrile), and 10 μl samples were injected and isocratic runs were conducted for 15 min in 100% running solvent at a flow rate of 1.0 ml/min. We identified AFB using a fluorescent detector (Waters) with excitation and emission wavelengths of 365 and 455 nm, respectively. AFB production for each strain was assessed using five flasks, and each experiment was repeated three times.

### Microscopic Examination of AflGcnE-mCherry Subcellular Localization

The *A. flavus* AflGcnE-mCherry strains were prepared using a modified published procedure (Wong et al., 2008) and the primers listed in **Supplementary Table S3**. To construct the *AflgcnE*-mCherry fragment, four separate fragments were amplified by PCR. The *gcnE*-P1 and *gcnE*-P2 primers were used to amplify the *gcnE* open reading frame (ORF) lacking the termination codon (TAG). The mCherry-P3 and mCherry-P4 primers were used to amplify the mCherry sequence carrying the TAG termination codon, while the *pyrG*-P5 and *pyrG*-P6 primers were used to amplify the selection marker. A region downstream of *gcnE* was amplified with primers *gcnE*/P7 and *gcnE*/P8. The *AflgcnE*-mCherry-P9 and *AflgcnE*-mCherry-P10 primers were used to combine the *gcnE* ORF, mCherry sequence, *pyrG* selection marker, and the sequence downstream of *gcnE*. The purified *AflgcnE*-mCherry fragments were inserted into PTSΔ*ku70*Δ*pyrG* protoplasts as described above. To assess AflGcnE-mCherry localization, fresh conidia and mycelia were analyzed using the Leica confocal SP8 microscope. The nuclei of mycelia and conidia were observed after samples were stained with 1 μg/ml 4′,6-diamidino-2-phenylindole (DAPI, Sigma, USA).

### Maize Seed Colonization Assay

A maize seed colonization assay was completed using a modified published procedure (Kale et al., 2008). A mycelial plug for each strain was grown in 1.5 ml YES liquid medium overnight at 28°C with shaking (180 rpm). Twenty surface-sterilized maize (*Zea mays*) seeds were added to the overnight cultures, and samples were incubated at 37°C for 30 min with shaking (50 rpm). The control sample (i.e., mock inoculation) consisted of maize seeds in sterile water. The maize seeds were incubated at 28°C on filter paper, which was moistened daily to maintain humidity. Maize seeds were harvested in 50-ml Falcon tubes after a 7-day incubation. The seeds were then vigorously mixed in 20 ml sterile water supplemented with 0.05% Tween-80 for 2 min to release the spores into the solution. We counted the number of conidia as described earlier. To extract AFB1 from maize seeds, 10 ml acetone was added to each tube, which was then shaken at 150 rpm for 10 min. Samples were incubated at room temperature for another 10 min and then centrifuged at 2,000 rpm for 15 min. The organic layer was transferred to a new tube and dried completely in a fume hood. Samples were resuspended in a solution consisting of 2.5 ml hexane and 5 ml 0.1 M NaCl in methanol:water (55:45), vortexed for 1 min, and centrifuged at 2,000 rpm for 5 min. The hexane phase was added to a new plate, while the fatty acid interphase layer was discarded. The remaining aqueous phase was treated with hexane two more times and collected. The hexane samples were combined, dried completely, and resuspended in 500 μl chloroform. A 10-μl aliquot of each extract was separated on a TLC plate using a chloroform:acetone (95:5) solvent system. The TLC plate was exposed to UV light and photographed as described earlier. Each experiment was repeated three times.

### Lipase Activity Assay

Lipase activity was analyzed using a modified published method (Amaike et al., 2013). Each strain was cultured on YES agar medium for 2 days, after which mycelial plugs were used to inoculate 8 ml tributyrin agar medium (0.3% yeast extract, 0.5% peptone, 0.1% tributyrin, 1% agar, pH 7.5) in 10-ml test tubes. The strains were grown at 28°C in darkness, and the clearing zones were measured from day 3 to day 7. Each experiment was repeated twice with five replicates each.

### Quantitative Reverse Transcription Polymerase Chain Reaction

To determine *gcnE* expression levels in different developmental stages, *A. flavus* NRRL3357 mycelia were grown in YES broth at 37°C and collected after 24, 48, 72, and 144 h. To analyze the expression level of sporulation-related genes, mycelia were collected from YES agar medium after cultures were incubated at 37°C for 48 and 72 h. To examine the expression level of genes related to sclerotial development, mycelia were grown on Wickerham agar medium at 37°C and harvested after 7 days. The expression of aflatoxin-related genes was assessed using mycelia collected from YES broth incubated at 28°C for 48 and 72 h. The collected mycelia were ground in liquid nitrogen. Total RNA was isolated from approximately 50 mg ground mycelia for each strain using an RNA isolation kit (Promega, USA). For quantitative RT-PCR (qRT-PCR) analysis, cDNA was synthesized from 2 μg RNA using the Revert Aid First-strand cDNA Synthesis kit (Thermo Fisher Scientific). The qRT-PCR was completed using the SYBR Green Supermix (Takara) and the PikoReal 96 Real-time PCR system (Thermo Fisher Scientific). The relative quantities of each transcript were calculated using the 2^-ΔΔCT^ method (Livak and Schmittgen, 2001). All transcript levels were normalized relative to that of the *β-tubulin* housekeeping gene. The qRT-PCR primers are listed in **Supplementary Table S4**. All qRT-PCR analyses were completed in triplicate, and each experiment was repeated three times.

### Western Blot Analysis

Mycelia of each strain were collected from YES broth incubated at 28°C for 72 h. Samples were frozen in liquid nitrogen and ground to a fine powder for subsequent protein extractions. Approximately 100 mg ground powder was resuspended in 1 ml radio immunoprecipitation assay lysis buffer (RIPA, Beyotime, Shanghai, China), and whole proteins were extracted according to the manufacturer’s instruction. Equal amounts of proteins were separated by 15% sodium dodecyl sulfate polyacrylamide gel electrophoresis and transferred to a polyvinylidene fluoride membrane (Millipore, USA) using an electroblotting apparatus (Bio-Rad, USA). Histone modifications were detected with the anti-acetyl-histone H3 (1:2,000 dilution; Millipore), anti-acetyl-histone H3K9 (1:2,000 dilution; PTM BioLabs, Hangzhou, China), anti-acetyl-histone H3K14 (1:2,000 dilution; PTM BioLabs), and anti-histone H3 (1:750 dilution; Abcam, UK) antibodies. A horseradish peroxidase-conjugated goat anti-rabbit antibody was used as the secondary antibody (1:10,000 dilution; Abgent, USA). The WesternBright^TM^ Quantum chemiluminescent HRP substrate was used (Advansta, USA), and chemiluminescence was detected using the Gene Imaging System (Syngene, Hong Kong, China).

### Statistical Analysis

Data were presented as means ± standard deviation of at least three biological replicates samples in figures. Statistical and significance analysis were performed using the GraphPad Prism 5 and regarded significant if *p*-values were <0.05. Student’s *t*-test was used when comparing two means for differences. For multiple comparisons, Tukey’s multiple comparison test was used for significance analysis.

## Results

### Identification *Aspergillus flavus* GcnE

Putative *A. flavus* Gcn5 homologs were identified by searching the NCBI database (see text footnote 1) with a basic local alignment search tool (BLAST) algorithm using the *S. cerevisiae* Gcn5 protein (GenBank accession number: AJS29493.1) as a query. An *A. flavus* protein designated as GcnE (GenBank accession number: AFLA_051420) was 65 and 43% similar to the corresponding *S. cerevisiae* and *H. sapiens* genes, respectively. The *A. flavus gcnE* ORF consists of 1,530 bp with six introns, and encodes a putative histone acetylase with 402 amino acids. Similar to *S. cerevisiae* Gcn5, AflGcnE harbors a GCN5-related N-acetyltranferase (GNAT) domain (residues 66–236) and a C-terminal bromodomain (residues 276–400) that are 80 and 56% identical to the corresponding *S. cerevisiae* Gcn5 domains, respectively (**Supplementary Figure S1A**). The Gcn5 amino acid sequences from several species (14 fungi, one plant, and three animals) were downloaded from the NCBI database, and analyses of the protein domains indicated that all of the analyzed Gcn5 proteins share a highly conserved structure consisting of a GNAT domain and a DNA-binding C-terminal bromodomain (**Supplementary Figure S1A**). The amino acid sequence is conserved among Gcn5 and its orthologs in fungi, plants, and animals (**Supplementary Figure S2**). The phylogenetic tree constructed based on Gcn5 amino acid sequences revealed that *A. flavus* GcnE is 100% identical to its homolog in the important industrial fungi *Aspergillus oryzae*, and 95% identical to its homolog in the related model species *A. nidulans*. Gcn5 exists in organisms from eukaryotic fungi to mammals (**Supplementary Figure S1B**), suggesting Gcn5 is crucial for survival.

### Analysis of *AflgcnE* Expression and Construction of the Δ*AflgcnE* Mutant and Δ*AflgcnE-C* Complemented Strain

To analyze *AflgcnE* expression levels in the vegetative and stationary phases of the *A. flavus* lifecycle, we determined the *gcnE* transcription level at 24, 48, 72, and 144 h (**Supplementary Figure S3A**). The *AflgcnE* transcription level was relatively unchanged before 72 h, but increased after 144 h (**Supplementary Figure S3A**), implying *AflgcnE* may affect the vegetative and stationary phases in *A. flavus*. To clarify the *AflgcnE* functions, we deleted the gene using a homologous recombination strategy as previously described (Yang et al., 2016; **Supplementary Figure S3B**). The selected Δ*AflgcnE* transformants were analyzed by PCR (**Supplementary Figure S3C**), and the resulting PCR products (UA and DA in **Supplementary Figure S3B**) were verified by DNA sequencing (data not shown). To confirm that the phenotypic changes observed in Δ*AflgcnE* were due to the deletion of *AflgcnE*, the Δ*AflgcnE* mutant protoplast was complemented with a pPTRI plasmid containing full-length *AflgcnE*. Selected Δ*AflgcnE-C* complemented strains were also confirmed by PCR using genomic DNA as the template. All of the selected strains were analyzed by RT-PCR, which confirmed that *AflgcnE* transcripts were undetectable in the Δ*AflgcnE* mutant, in contrast to the WT and Δ*AflgcnE-C* strains (**Supplementary Figure S3D**). These results indicate that the Δ*AflgcnE* mutant and Δ*AflgcnE-C* complemented strains were successfully constructed.

### Δ*AflgcnE* Decreases the Acetylation of Histones H3 and H3K14

**FIGURE 1:**
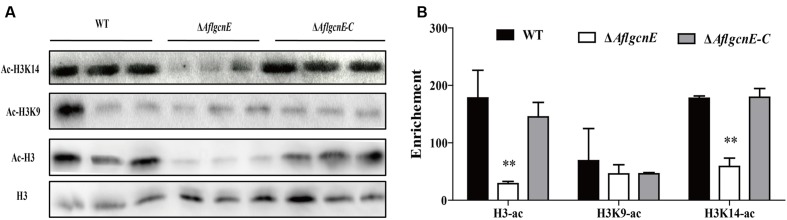
**Western blotting analysis of proteins that extracted from WT, Δ*AflgcnE*, and Δ*AflgcnE-C* strains, respectively.** **(A)** The antibody anti-acetyl histone 3 (ac-H3), anti-acetyl H3K9 (ac-H3K9), anti-acetyl H3K14 (ac-H3K14) were performed for acetylation analysis, antibody H3 was used as a loading reference. **(B)** Enrichment levels of **(A)** by the bank optical density assay. ^∗∗^*p* < 0.01.

Gcn5 is responsible for the acetylation of histone H3 in many fungi (Vogelauer et al., 2000; Nützmann et al., 2011). Therefore, we examined the acetylation of histone H3 and its canonical targets, H3K9 and H3K14, in *A. flavus*. Histones H3 and H3K14 were acetylated more in the WT and Δ*AflgcnE-C* strains than in the Δ*AflgcnE* mutant (**Figure 1**). Surprisingly, there was no difference in the acetylation of H3K9 between the WT, Δ*AflgcnE*, and Δ*AflgcnE-C* strains. These results confirmed that AflGcnE is a HAT, and its target in *A. flavus* is H3K14.

### Subcellular Localization of AflGcnE in *Aspergillus flavus* Depends on the Developmental Stage

**FIGURE 2:**
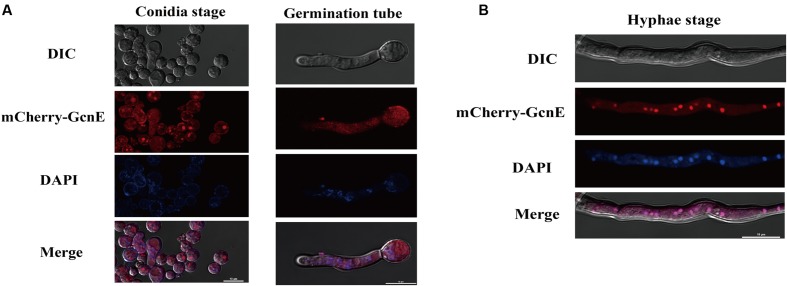
**Subcellular localization of AflGcnE-mCherry in *A. flavus*.** **(A)** Localization of AflGcnE-mCherry in conidial stage, and germination stage, images were shown confocal differential interference contrast (DIC) imaging, the localization of AflGcnE-mCherry, stained with the nucleus marker DAPI, and merge photo of nucleus and AflGcnE-mCherry. Germination spores were collected after cultured in YES liquid media for 6 h at 37°C. **(B)** Localization of AflGcnE-mCherry in hyphal stages, mycelia were collected from YES liquid media after grown at 37°C for 24 h, bars = 10 μm.

We investigated the subcellular localization of AflGcnE using a previously described method (Wong et al., 2008). We constructed a strain expressing the mCherry tag at the C-terminal of AflGcnE under the control of the native promoter (AflGcnE-mCherry). The AflGcnE-mCherry strains exhibited the same phenotype as the CA14 strain, suggesting the mCherry tag did not affect GcnE function (data not shown). In germinating spores cultured in YES liquid medium for 6 h, AflGcnE-mCherry mainly accumulated in the cytoplasm and nucleus (**Figure 2A**). In mycelia collected from YES liquid medium at 24 h (i.e., mycelial growth stage), a strong fluorescent signal was mainly observed in nuclei (**Figure 2B**).

### *AflgcnE* Influences Vegetative Growth and Cell-Surface Hydrophobicity

**FIGURE 3:**
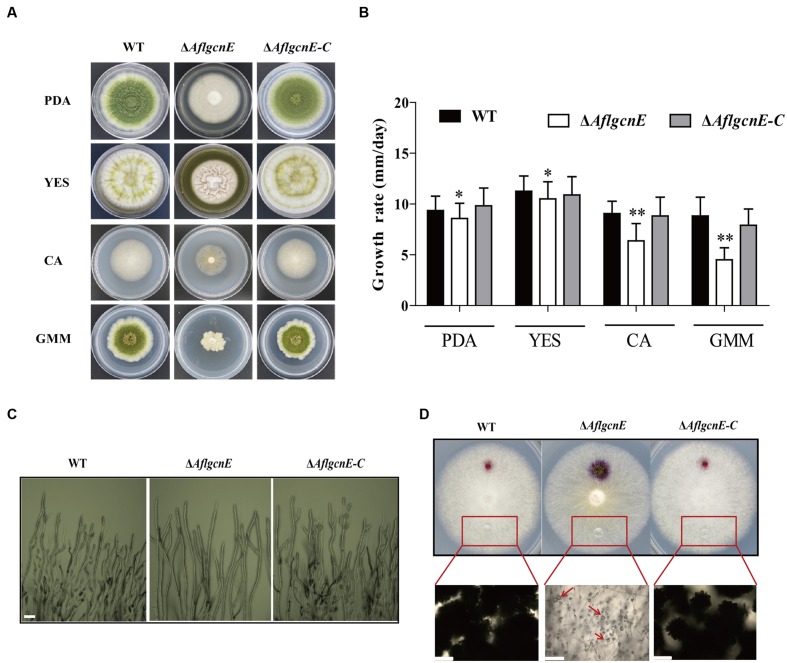
**Phenotype, growth rate, mycelial branches, and hydrophobicity analysis of WT, Δ*AflgcnE*, and Δ*AflgcnE-C* strain.** **(A)** Phenotype of WT, Δ*AflgcnE*, and Δ*AflgcnE-C* strains, after grown on PDA, YES, CA, and GMM media at 37°C for 5 days, respectively. **(B)** Growth rate of WT, Δ*AflgcnE*, and Δ*AflgcnE-C* strains. **(C)** Microscopic examination revealed the different mycelial tips of WT, Δ*AflgcnE*, and Δ*AflgcnE-C* strains, bars = 100 μm. **(D)** Hydrophobicity assay of WT, Δ*AflgcnE*, and Δ*AflgcnE-C* strains, red arrows indicate the absorbed water in the colony, bars = 100 μm, ^∗^*P* < 0.05, ^∗∗^*P* < 0.01.

The production of aerial hyphae was relatively low for the Δ*AflgcnE* mutant on nutrient-rich and minimal media, including PDA, YES, CA, and GMM (**Figure 3A**). Additionally, the growth rate of the Δ*AflgcnE* mutant was considerably lower than that of the WT and Δ*AflgcnE-C* strains in PDA (*p* < 0.05) and YES (*p* < 0.05) media (i.e., nutrient-rich media), as well as in CA medium (*p* < 0.01) and GMM (*p* < 0.01; **Figure 3B**). Microscopic examinations indicated that the Δ*AflgcnE* mutant generated less mycelia and fewer branches at the mycelial tips compared with the WT and Δ*AflgcnE-C* strains (**Figure 3C**).

A hydrophobic cell surface contributes to hyphal formation and is a distinguishable feature of aerial hyphae (Kershaw and Talbot, 1998). The inhibited production of aerial hyphae in the Δ*AflgcnE* mutant may be associated with decreased hydrophobicity at the cell surface. To confirm this, the surface of each fungal strain cultured on CA medium was treated with 20 μl water. The Δ*AflgcnE* mutant exhibited a wettable phenotype, with the water spreading and being absorbed (**Figure 3D**). In contrast, the water generated spherical droplets on the WT and Δ*AflgcnE-C* colonies. These differences were more easily visualized when the applied water was supplemented with 2.5% bromophenol blue, and suggest the Δ*AflgcnE* strain was defective regarding cell-surface hydrophobicity (**Figure 3D**).

### *AflgcnE* Is Essential for Conidial Formation

**FIGURE 4:**
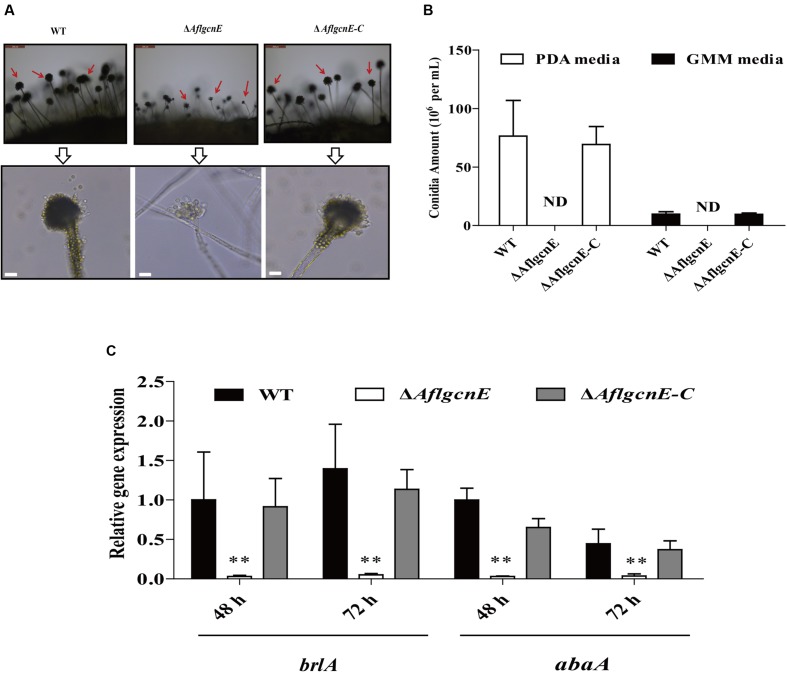
**The Δ*AflgcnE* strain was defected in conidiation.** **(A)** Conidiophores was observed under a light microscope, after induction with illumination for 12 h, bars = 50 μm. **(B)** Deletion of *AflgcnE* resulted in absence of conidia, the abbreviation ND represents no detection of conidia. **(C)** qRT-PCR analysis of conidial formation relevant gene *brlA* and *abaA*, after being cultured on PDA at 37°C for 48 and 72 h, respectively. All transcription levels were normalized to *β-tubulin* as the house keeping gene, and calculated by 2^-ΔΔCT^ method. ^∗^*P* < 0.05, ^∗∗^*P* < 0.01.

Conidiophores and conidial formation were analyzed to char acterize the role of *AflgcnE* during reproduction. Microscopic examinations revealed that the Δ*AflgcnE* mutant produced shorter stalks and fewer conidiophores than the WT and Δ*AflgcnE-C* strains (**Figure 4A**). Additionally, the conidial heads in the Δ*AflgcnE* mutant remained immature, and the phialides did not generate conidial chains or produce conidia (**Figure 4A**). Further quantitative analysis of conidial formation confirmed that the Δ*AflgcnE* mutant was unable to form conidia in PDA or GMM, in contrast to the WT and Δ*AflgcnE-C* strains (**Figure 4B**). Furthermore, the expression of *brlA* and *abaA*, which regulate conidial formation, was down-regulated in the Δ*AflgcnE* mutant cultured for 48 and 72 h (**Figure 4C**).

### *AflgcnE* Is Essential for Sclerotial Generation

**FIGURE 5:**
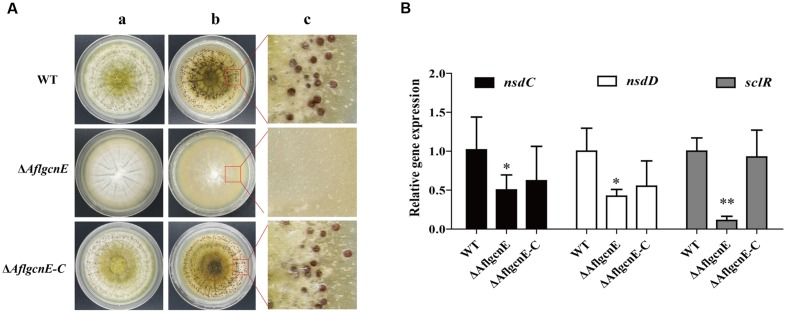
**Sclerotial formation analysis between WT, Δ*AflgcnE*, and Δ*AflgcnE-C* strains.** **(A)** Phenotype of WT, Δ*AflgcnE*, and Δ*AflgcnE-C* strains on WKM media, after grown for 7 days at 37°C, the red arrows indicates development of conidia head between different strains, (a) before ethanol washed; (b) after washed by ethanol; (c) detail views of sclerotia. **(B)** Transcriptional expression levels of *nsdC*, *nsdD*, and *sclR*. ^∗^*P* < 0.05; ^∗∗^*P* < 0.01. All transcription levels were normalized to *β-tubulin* as the house keeping gene, and calculated by 2^-ΔΔCT^ method.

*Aspergillus flavus* generates sclerotia, which are resting bodies that enable the fungus to survive in unsuitable environments. In addition to the defects observed in conidial formation, the Δ*AflgcnE* mutant did not produce sclerotia on the sclerotia-conducive Wickerham medium (**Figure 5A**). In contrast, the WT strain generated sclerotia (30.98 ± 3.62 mg/plate), as did the Δ*AflgcnE-C* strain (28.39 ± 1.74 mg/plate). Furthermore, the expression levels of the following genes related to sclerotial production were lower in the Δ*AflgcnE* mutant than in the WT and Δ*AflgcnE-C* strains: *nsdC* (AFLA_131330), *nsdD* (AFLA_020210), and *sclR* (AFLA_040260; **Figure 5B**).

### *AflgcnE* Is Required for Maintaining Cell Wall Integrity and Genotoxic Stress Responses

**FIGURE 6:**
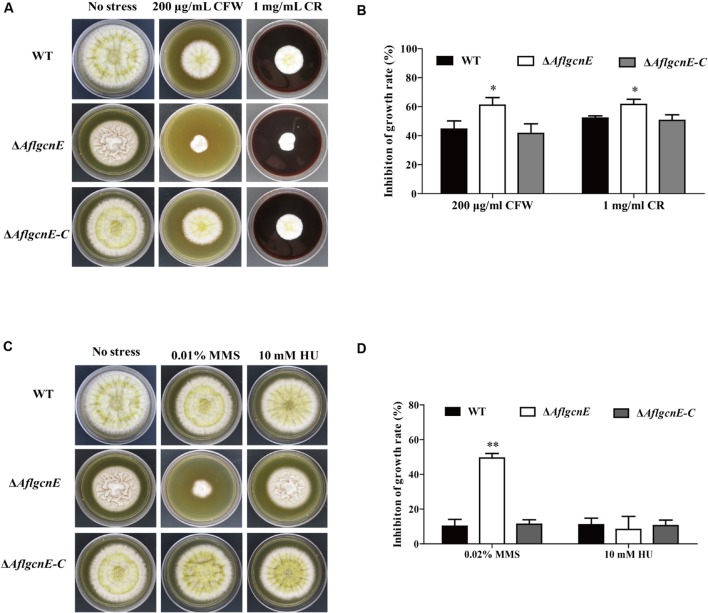
**Phenotype and inhibition growth rate of WT, Δ*AflgcnE*, and Δ*AflgcnE-C* strains under cell wall integrity stress and genomic integrity stress.** **(A)** Morphology of WT, Δ*AflgcnE*, and Δ*AflgcnE-C* strains under cell wall stress generated by 200 μg/ml CFW and 1 mg/ml CR, after 5 days at 37°C, inhibition of growth rate was relative to the growth rate of each untreated strain [inhibition of growth rate = (the diameter of untreated strain - the diameter of treated strain)/(the diameter of untreated strain) × 100%]. **(B)** Growth inhibition of WT, Δ*AflgcnE*, and Δ*AflgcnE-C* strains under cell wall stress. **(C)** Morphology of WT, Δ*AflgcnE*, and Δ*AflgcnE-C* strains under genotoxic stress generated by 0.02% MMS and 10 mM HU. **(D)** Growth inhibition of WT, Δ*AflgcnE*, and Δ*AflgcnE-C* strains under genomic integrity stress. ^∗^*P* < 0.05; ^∗∗^*P* < 0.01.

We evaluated the sensitivity of WT, Δ*AflgcnE*, and Δ*AflgcnE-C* strains to cell wall, osmotic, genotoxic, and oxidative stresses. The Δ*AflgcnE* mutant was more sensitive to the cell wall stress induced by calcofluor white and Congo red than the WT and Δ*AflgcnE-C* strains (**Figures 6A,B**). The Δ*AflgcnE* mutant was also more sensitive to the genotoxic stress generated by methyl methanesulfonate than the other two strains. However, there were no growth rate differences between the WT, Δ*AflgcnE*, and Δ*AflgcnE-C* strains under the genotoxic stress conditions produced by hydroxyurea (**Figures 6C,D**). Additionally, the sensitivity of the Δ*AflgcnE* mutant to osmotic and oxidative stresses did not differ from that of the WT and Δ*AflgcnE-C* strains (**Supplementary Figure S4**). These results indicate that *AflgcnE* is involved in responses to cell wall and genotoxic stresses, but not to hyperosmotic or oxidative stresses.

### *AflgcnE* Regulates Aflatoxin Production

**FIGURE 7:**
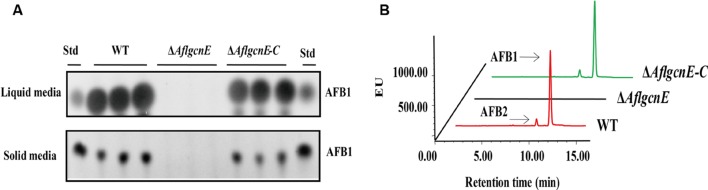
**Requirement of *AflgcnE* for aflatoxin biosynthesis.** **(A)** TLC results showed the absence of AFB1 production in Δ*AflgcnE* strains in both YES liquid and solid media after grown at 28°C for 72 h, abbreviation Std represent the AFB1 standard. **(B)** HPLC profiles showed that the Δ*AflgcnE* strains did not produce AFB1, as well as AFB2 production.

A previous study concluded that *A. nidulans gcnE* mediates the synthesis of secondary metabolites (Nützmann et al., 2011). Thus, we investigated the production of aflatoxins, which are the most crucial and abundant secondary metabolites in *A. flavus*. The TLC results indicated that the Δ*AflgcnE* mutant was unable to produce AFB1, whereas AFB1 production was observed for the WT and Δ*AflgcnE-C* strains (**Figure 7A**). The HPLC profiles confirmed the lack of AFB1 and AFB2 production in the Δ*AflgcnE* strain (**Figure 7B**). The consequences of the absence of AflgcnE on aflatoxin synthesis was further analyzed by qRT-PCR. The expression of aflatoxin-specific regulatory genes (i.e., *aflR* and *aflS*) was lower in the Δ*AflgcnE* mutant than in the other two strains (**Table 1**). Similarly, the gene expression levels of the early-expressed structural genes, *aflC* (*pksA*) and *aflD* (*nor-1*), were considerably lower in the Δ*AflgcnE* mutant (**Table 1**). Furthermore, expression of the mid- and late-expressed genes related to aflatoxin biosynthesis [i.e., *aflK* (*vbs*), *aflO* (*OmtA*), *aflP* (*OmtB*), and *aflQ* (*ordA*)] was almost undetectable in the Δ*AflgcnE* mutant (**Table 1**).

**Table 1:** qRT-PCR analysis of the aflatoxin biosynthesis genes in WT, Δ*AflgcnE*, and Δ*AflgcnE-C* strains.

Accession number	Gene name	Relative expression level (48 h)			Relative expression level (72 h)		
			
		WT	Δ*AflgcnE*	Δ*AflgcnE-C*	WT	Δ*AflgcnE*	Δ*AflgcnE-C*
AFLA_139360	*aflR*	1.00 ± 0.04	0.27 ± 0.06^∗∗^	1.28 ± 0.40	1.00 ± 0.02	0.17 ± 0.07^∗∗^	0.88 ± 0.29
AFLA_139340	*aflS*	1.00 ± 0.06	0.12 ± 0.03^∗∗^	0.86 ± 0.24	1.00 ± 0.16	0.22 ± 0.03^∗∗^	1.35 ± 0.14
AFLA_139410	*aflC*	1.00 ± 0.00	0.10 ± 0.06^∗∗^	1.55 ± 0.66	1.00 ± 0.08	0.03 ± 0.06^∗∗^	0.75 ± 0.26
AFLA_139390	*aflD*	1.00 ± 0.01	0.03 ± 0.01^∗∗^	1.13 ± 0.40	1.00 ± 0.05	0.02 ± 0.01^∗∗^	0.63 ± 0.40
AFLA_139190	*aflK*	1.00 ± 0.02	0.009 ± 0.003^∗∗^	1.05 ± 0.33	1.00 ± 0.06	0.007 ± 0.003^∗∗^	1.46 ± 0.33
AFLA_139220	*aflO*	1.00 ± 0.00	0.0005 ± 0.0006^∗∗^	1.14 ± 0.34	1.00 ± 0.09	0.01 ± 0.03^∗∗^	1.75 ± 0.26
AFLA_139210	*aflP*	1.00 ± 0.00	0.0006 ± 0.0003^∗∗^	1.39 ± 0.44	1.00 ± 0.06	0.0007 ± 0.0001^∗∗^	1.68 ± 0.40
AFLA_139200	*aflQ*	1.00 ± 0.06	0.05 ± 0.02^∗∗^	1.12 ± 0.15	1.00 ± 0.04	0.10 ± 0.02^∗∗^	1.52 ± 0.15


*All transcription levels were normalized to β-tubulin as the house keeping gene, and calculated by 2^-ΔΔ*CT*^ method. ^∗∗^*p* < 0.01.*

### Maize Seed Colonization Is Impaired in the Δ*AflgcnE* Mutant

**FIGURE 8:**
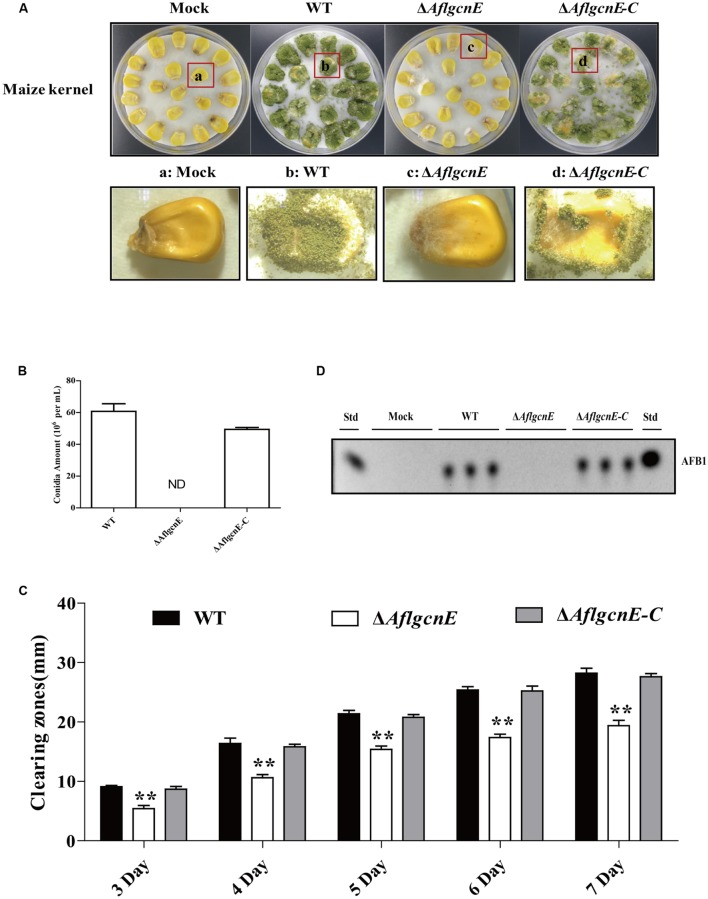
***AflgcnE* is required for infecting maize seeds.** **(A)** Photographs presented the infected maize seeds with WT, Δ*AflgcnE*, and Δ*AflgcnE-C* strains after incubation at 28°C for 7 days. **(B)** Quantification of conidia from the infected maize seeds by each strain, abbreviation ND represented no detection of conidia. **(C)** TLC results showed the AFB1 production extracted from infected seeds by WT, Δ*AflgcnE*, and Δ*AflgcnE-C* strains. **(D)** The WT, Δ*AflgcnE*, and Δ*AflgcnE-C* strains were grown on tributyrin agar media, and zones of clearing were measured from 3 to 7 days incubation at 28°C in dark condition. ^∗∗^*P* < 0.01.

To examine the role of *gcnE* in the interaction between *A. flavus* and seeds, as well as the resulting pathogenesis, we evaluated the ability of the Δ*AflgcnE* mutant to colonize maize seeds. Surface-sterilized viable maize seeds were inoculated with WT, Δ*AflgcnE*, and Δ*AflgcnE-C A. flavus* strains. The Δ*AflgcnE* mutant was less able to colonize maize seeds compared with the other two strains (**Figure 8A**). We also observed a lack of conidiation in the Δ*AflgcnE* mutant-infected maize seeds, which was in contrast to the seeds infected with the WT and Δ*AflgcnE-C* strains (**Figure 8B**). Additionally, AFB1 was not detected in maize seeds infected with the Δ*AflgcnE* mutant (**Figure 8C**). The ability of *A. flavus* to colonize seeds is associated with lipase activity (Brown et al., 2009; Dolezal et al., 2013). Based on the observations of Δ*AflgcnE* mutant-infected maize seeds, we hypothesized that lipase activity is impaired in the mutant strain. Each strain was cultured on a medium containing tributyrin, which is a short-chain fatty acid. The clearing zones surrounding the Δ*AflgcnE* mutant colony were smaller than those surrounding the WT and Δ*AflgcnE-C* strains at all time-points (**Figure 8D**), indicating lipase activity was lower in the Δ*AflgcnE* mutant.

## Discussion

Interestingly, our results indicate that *A. flavus* AflGcnE localizes to the nucleus and cytoplasm during the conidial production and germination stages, whereas it is present mainly in the nucleus (**Figure 2**) during the mycelial development stage. As a HAT, GcnE is generally believed to be localized to the nucleus. This is supported by the fact a previous study confirmed that *C. neoformans* Gcn5 is located in the nucleus (O’Meara et al., 2010). However, Gcn5 may regulate cellular functions partially through modifications of non-histone substrates in the cytoplasm. For example, Gcn5 is responsible for acetylating Ifh1 (i.e., transcription factor) in yeast (Downey et al., 2013). Consistent with our GcnE subcellular localization results, a previous study revealed that *Schizosaccharomyces pombe* Gcn5 is present in the nucleus and cytoplasm (Zhou et al., 2014). Additionally, *C. albicans* Gcn5 is distributed in the cytoplasm and nucleus during the vegetative growth stage, while it accumulates in the nucleus during the stationary phase (Chang et al., 2015). The *S. cerevisiae* Gcn5 moves to the cytoplasm in response to hypoxic conditions (Dastidar et al., 2012). These findings support the notion that Gcn5 is associated with diverse substrates during growth, and is not active only in the nucleus.

The importance of Gcn5 for morphogenesis has been broadly studied in fungal species. For example, a mutation to *gcnE* in *A. nidulans* results in the formation of immature and aberrant conidiophores (Cánovas et al., 2014). In *Trichoderma reesei*, the null *gcn5* mutant strain does not sporulate, and its hyphal cells are shorter and more swollen than those of the WT strain (Xin et al., 2013). Additionally, defects in hyphal development always result in dimorphic fungi, as observed for *C. albicans* (Chang et al., 2015), *C. neoformans* (O’Meara et al., 2010), and *U. maydis* (González-Prieto et al., 2014). However, the growth rate of the *S. cerevisiae gcn5* null mutant does not differ from that of the WT strain, except under conditions of limited NH_4_NO_3_ concentrations (Georgakopoulos and Thireos, 1992). In contrast to our results, the *Phytophthora sojae gcn5* mutant develops normally, but is hypersensitive to hydrogen peroxide (Zhao et al., 2015). These observations imply that Gcn5 regulates morphogenesis in a fungal species-specific manner. The results presented herein suggest that in addition to its effects on asexual development, AflGcnE is required for sclerotial production. Secondary metabolites are closely associated with fungal development (Calvo et al., 2002; Calvo and Cary, 2015). Our data revealed that the inability to produce AFB in the Δ*AflgcnE* mutant is related to inhibited expression of aflatoxin synthesis genes, including *aflD* (*pksA*). However, a previous study revealed that the deletion of *pksA* leads to increased sclerotial formation (Mahanti et al., 1996). Our data indicate the lack of sclerotial production might be associated with the expression of sexual development-related genes such as *nsdC*, *nsdD*, and *sclR* (**Figure 5**). Further research is required to fully characterize the relationship between GcnE and sclerotial formation.

Our findings suggest that GcnE influences cell wall integrity and genome stability. This is consistent with previous studies that concluded that *C. albicans* Gcn5 (Chang et al., 2015) and *C. neoformans* Gcn5 (O’Meara et al., 2010) affect cell wall integrity. Genes that regulate cell wall biosynthesis are usually also involved in cell wall integrity signaling. Treatments with calcofluor white result in decreased β-glucan accumulation, while exposure to Congo red lowers the cell wall chitin content (Hagiwara et al., 2011). The effects of GcnE on *A. flavus* cell wall integrity may be due to its regulation of the oligosaccharyltransferase Stt3 or the cell wall chitin accumulation factor Smp1. Gcn5 binding is enriched for these stress-related genes in *S. cerevisiae* (Huisinga and Pugh, 2004). Furthermore, our results regarding the decreased cell-surface hydrophobicity of the Δ*AflgcnE* mutant (**Figure 3**) are in agreement with those of an earlier study that observed that decreased cell-surface hydrophobicity is associated with altered cell wall composition (Kershaw and Talbot, 1998). Our observations also confirm that GcnE-mediated transcriptional regulation is required to maintain genomic integrity. This is not surprising because researchers have previously reported that GcnE mediates histone H3 acetylation, which affects chromatin assembly and ultimately stabilizes the genomic integrity (Abate et al., 2012).

Aflatoxin biosynthesis is associated with responses to oxidative stress (Grintzalis et al., 2014). We observed a lack of AFB accumulation in the Δ*AflgcnE* mutant (**Figure 7**). There was no difference between the Δ*AflgcnE* mutant and WT strain regarding responses to the oxidative stress generated by hydrogen peroxide and *tert*-butyl hydroperoxide. This is consistent with the fact *C. albicans* Gcn5 is not involved in oxidative stress responses (Chang et al., 2015). However, some fungal species are more sensitive to oxidative stress than *A. flavus*, including *C. neoformans* (O’Meara et al., 2010) and *P. sojae* (Zhao et al., 2015). As a core subunit of the SAGA complex, Gcn5 affects the yeast HOG pathway (Spedale et al., 2012), but Hog1 kinase does not appear to be involved in *A. flavus* responses to oxidative and osmotic stresses (Baidya et al., 2014). Alternatively, Gcn5 may regulate responses to these stresses in a species-specific manner. The exact mechanism remains to be determined.

In many fungi, modifications due to acetylation are related to the production of secondary metabolites. The extent of the acetylation of histone H4 in the *aflR* promoter region affects the production of AFB in *A. parasiticus* (Roze et al., 2007). In *T. reesei*, cellulase production is considerably decreased in the *gcn5* null mutant, which is associated with decreased acetylation of histone H3 in the *cbh1* promoter region (Xin et al., 2013). Similarly, sterigmatocystin and terrequinone production in *A. nidulans* is influenced by the acetylation of histone H3 in the promoter regions of *aflR* and *stcO* (for sterigmatocystin) and *tdiA* and *tdiB* (for terrequinone; Nützmann et al., 2011). The Δ*AflgcnE* mutant was unable to synthesize aflatoxin. We also observed that GcnE affected the expression of genes related to AFB biosynthesis, which implies GcnE facilitates AFB biosynthesis in *A. flavus*. Unlike a previous *S. cerevisiae* study that described a considerable decrease in the acetylation of H3K9 in a *gcn5* mutant (Islam et al., 2011), we did not observe any differences in H3K9 acetylation in *A. flavus* (**Figure 1**). There is a rational explanation for this discrepancy. For example, in *A. nidulans*, the synthesis of secondary metabolites is accompanied by increased acetylation of H3K14, whereas increased acetylation of H3K9 occurs only within gene clusters (Nützmann et al., 2011). Further study revealed the acetylation of H3K14 is more important than the acetylation of H3K9 in *A. nidulans* (Nützmann et al., 2013). Taken together, we hypothesize that AflGcnE is required for inducing aflatoxin biosynthesis *via* the acetylation of H3K14 in the promoter regions of aflatoxin genes. However, the possible involvement of acetylated H3K9 requires further investigation.

The importance of acetylation-induced modifications in fungal pathogens has been well known for many years. Among human pathogens, the *C. neoformans gcn5* null mutant is avirulent in an animal model of cryptococcosis (O’Meara et al., 2010). Additionally, Gcn5 is required for the pathogenesis of *C. albicans* in a mouse model (Chang et al., 2015). Similarly, among plant pathogens, the *U. maydis gcn5* mutant is avirulent to maize plants (González-Prieto et al., 2014), while *P. sojae* Gcn5 is required for full virulence during infections of soybean (Zhao et al., 2015). We observed that the ability of the Δ*AflgcnE* mutant to infect maize seeds is inhibited (**Figure 8**), confirming the importance of Gcn5 for the pathogenicity of this species. During seed colonization, *A. flavus* produces diverse extracellular hydrolytic enzymes, such as lipases, to acquire nutrients from the host. Lipase activities are associated with the virulence of fungal pathogens (Tsitsigiannis and Keller, 2006). The lipase activity level was lower in the Δ*AflgcnE* mutant than in the WT strain, suggesting the impaired virulence of the mutant may be due to decreased lipase activity. However, previous reports indicate that *A. flavus* virulence is regulated by several factors, including the global regulators LaeA and VeA (Amaike and Keller, 2009), the bZIP protein MeaB (Amaike et al., 2013), and the master transcription factor MtfA (Zhuang et al., 2016). Investigations of the relationships between GcnE and these factors regarding virulence are warranted.

In summary, we revealed that the HAT AflGcnE is necessary for *A. flavus* growth and development. It is also essential for asexual reproduction, conidial formation, sclerotial generation, and aflatoxin biosynthesis. Additionally, AflgcnE affects cell wall integrity, genotoxic stress resistance, and pathogenicity. These results indicate that the epigenetic modifications influencing the chromatin remodeling associated with AFB regulation are vital in filamentous fungi. AflGcnE may also represent a candidate target for controlling the contamination of crops by *A. flavus* AFBs.

## Author Contributions

HL, SW, and KY conceived and designed the experiments. HL, RS, and KF performed the experiments. HL and SW analyzed the data. HL, KY, FZ, XN, and SW wrote the paper. HL, ZZ, and SW originated research leading up to this paper and provided guidance and review.

## Conflict of Interest Statement

The authors declare that the research was conducted in the absence of any commercial or financial relationships that could be construed as a potential conflict of interest.

## Footnotes

1 http://www.ncbi.nlm.nih.gov/
