# Supplementary material for: The Aspergillus flavus Histone Acetyltransferase AflGcnE Regulates Morphogenesis, Aflatoxin Biosynthesis, and Pathogenicity
Source: Front Microbiol. 2016 Aug 30;7:1324. doi: 10.3389/fmicb.2016.01324 (PMC5003836; doi:10.3389/fmicb.2016.01324)
Supplement: Supplementary file 1 [file Data_Sheet_1.DOC]

**Supplemental information**

**Table S1 Fungal strains and plasmids used in this study.**

**Table S2 Specific-primers used in this study for *AflgcnE* gene knock-out and complement.**

**Table S3 Specific-primers used in this study for construct AflGcnE-mCherry strains.**

**Table S4 Specific-primers used in this study for qRT-PCR.**

**Figure S1. Expression analysis of *AflgcnE* and verification of Δ*AflgcnE* and Δ*AflgcnE-C* strains. (A).** Transcription level of *AflgcnE* at 24 h,48 h, 72 h and 144 h, strains were cultured in YES liquid media at 28℃ in dark condition, all transcription levels were normalized to *β-tublin* as the house keeping gene, and calculated by 2^−ΔΔCT^ method. **(B).** Graphic presentation present homologous recombination strategy to replace *AflgcnE* by a selective marker *pyrG* from *Aspergillus fumigatus*. The red box means the location of *AflgcnE*, the blue box means the selective marker gene *pyrG*, arrows and lines in purple were primers that used in this study to verify gene deletion. **(C).** Diagnostic PCR were performed to confirm the gene deletion and complemented strains, UA was confirmed by primers *gcnE*/UF and *gcnE*/UR, DA was confirmed by primers *gcnE*/DF and *gcnE*/DR. **(D).**Verification of gene deletion by RT-PCR analysis of *gcnE* expression in the fungi cultured in YES liquid media for 72 h, *β-tublin* gene was used as a reference.

**Figure S2. Domain and phylogenetic tree analysis of Gcn5. (A).** Domain analysis of Gcn5 from lower eukaryotic fungi to superior mammal animals, colour in grey means the unconserved regions, red box represents the GNAT domain, blue box represents DNA bromo domain, colour in yellow represents PCAF (P300/CBP-associated factor) domain. **(B).** A neighbor-joining phylogenetic tree was constructed by 18 species from *Asperillus* species to animals, scar bars=0.1.

**Figure S3. Alignment of amino acid sequences of Gcn5 and its putative orthologs from fungi to plant and human. Identical and similar residues are shaded in black and gray, respectively.**

Figure S4. Phenotype and inhibition growth rate of WT, Δ*AflgcnE* and Δ*AflgcnE-C* strains under osmotic stress and oxidative stress. (A).Morphology of WT, Δ*AflgcnE* and Δ*AflgcnE-C* strains under hyperosmotic stress generated by 1M NaCl and1M KCl, after 5 d at 37℃, inhibition of growth rate was relative to the growth rate of each untreated strain[Inhibition of growth rate=(The diameter of untreated strain-the diameter of treated strain)/(The diameter of untreated strain)×100%)]. (B).Growth inhibition under under hyperosmotic stress. (C). Morphologyunder oxidative stress generated by 0.5 mM tBOOH and 15mM H_2_O_2_. (D). Growth inhibitionunder oxidative stress, asterisk * represented p<0.05, asterisk ** represented p<0.01.

**Supplemental Tables**

**Table S1 Fungal strains and plasmids used in this study**

**Table 1 Fungal strains and plasmids used in this study**

| **Strains and plasmids name** | **Related gene type** | **Source** |
| --- | --- | --- |
| *Aspergillus flavus* NRRL3357 | Wild type | ([Zhang et al., 2015](#_ENREF_49)) |
| PTSΔ*ku70*Δ*pyrG* | Δ*ku70*,Δ*pyrG* | ([Chang et al., 2010](#_ENREF_1)) |
| PTSΔ*ku70* | Δ*ku70* | ([Chang et al., 2010](#_ENREF_1)) |
| *Aspergillus fumigatusAf293* | Wild type | ([Kong et al., 2013](#_ENREF_8)) |
| Δ*AflgcnE* | Δ*ku70*, Δ*gcnE::pyrG* | This study |
| Δ*AflgcnE-C* | Δ*ku70*,Δ*gcn5::pyrG*, *ptr-gcnE* | This study |
| *Escherichia coli* | *DH5α* | Takara, Japan |
| pPTR I | pPTR I | Takara, Japan |
| pPTR I-*gcnE* | pPTR I, +*AflgcnE* | This study |

**Table S2 Specific-primers used in this study for *AflgcnE* knockout andcomplement**

| **Primer name** | **Sequence(5’-3’)** | **Application** |
| --- | --- | --- |
| *gcnE*/AF | TTGATTCCTGTCTCTGATAAAGTGAT | Amplifying upstream |
| *gcnE*/AR | GGGTGAAGAGCATTGTTTGAGGCTCTTGTTAAGTGTACAAGTAAGTAAAGAGG |
| *gcnE*/BF | GCATCAGTGCCTCCTCTCAGACTCGATTGAATGATTGCATGAGCT | Amplifying downstream |
| *gcnE*/BF | CGAGGTTCCCTAAGGCATCAGA |
| *pyrG*/PF | GCCTCAAACAATGCTCTTCACCC | Amplifying selective marker |
| *pyrG*/PR | GTCTGAGAGGAGGCACTGATGC |
| *gcnE*/NF | GACGGTTGGACACTAACTGCC | Amplifying homologous flagments |
| *gcnE*/NF | TGTACGACGGCTGAGGACTTC |
| *gcnE*/UF | GCACCAGGAAGTTGTATCGT | Gene deletion screen |
| *gcnE*/UR | TCGGGAGCAGCGTAGAT |
| *gcnE*/DF | ATTGCTCGGCTACTTCAACTCA | Gene deletion screen |
| *gcnE*/DR | TGTGGAACAAGGGAAGACGATA |
| *gcnE*/CF | CGTGGTACCGGCTGGGTGTACGAGATA | Amplifying complemented flagments |
| *gcnE*/CR | GTAGGTACCGGTTGCGGGAGGGATTAG |

**Table S3 Specific-primers used in this study for construct AflGcnE-mCherry strains**

| **Primer name** | **Sequence(5’-3’)** | **Application** |
| --- | --- | --- |
| *gcnE*-P1 | ACGCCAAGATCCGTGCCT | Amplifying upstream |
| *gcnE*-P2 | CTCGCCCTTGCTCACCAT GTGGCCATCCGCCAGGT |
| *mCherry*-P3 | ATGGTGAGCAAGGGCGAG | Amplifying mCherry |
| *mCherry*-P4 | GGGTGAAGAGCATTGTTTGAGGCCTACTTGTACAGCTCGTCCAT |
| *pyrG*-P5 | GCCTCAAACAATGCTCTTCACCC | Amplifying selective marker |
| *pyrG*-P6 | GTCTGAGAGGAGGCACTGATGC |
| *gcnE* /P7 | GCATCAGTGCCTCCTCTCAGACTCGATTGAATGATTGCATGAGCT | Amplifying downstream |
| *gcnE* /P8 | CGAGGTTCCCTAAGGCATCAGA |
| *AflgcnE*-mCherry-P9 | GATCCGTTAAGTATCCCG | Nest primers for  *Aflgcn5*-mCherry |
| *AflgcnE*-mCherry-P10 | AGCAGCCGAAACTCCTA |

**Table S4 Specific-primers used in this study for qRT-PCR**

| **Primer name** | **Sequence(5’-3’)** | **Application** |
| --- | --- | --- |
| *gcnE*/QF | AACAACGAGAACACGCCTTA | qRT-PCR |
| *gcnE*/QR | TGCGACCACTCGGGAATA |
| *brlA/QF* | GCCTCCAGCGTCAACCTTC | qRT-PCR |
| *brlA/QR* | TCTCTTCAAATGCTCTTGCCTC |
| *abaA/QF* | CACGGAAATCGCCAAAGAC | qRT-PCR |
| *abaA/QR* | TGCCGGAATTGCCAAAG |
| *nsdC*/QF | GCCAGACTTGCCAATCAC | qRT-PCR |
| *nsdC*/QR | CATCCACCTTGCCCTTTA |
| *nsdD* /QF | GGACTTGCGGGTCGTGCTA | qRT-PCR |
| *nsdD* /QR | AGAACGCTGGGTCTGGTGC |
| *sclR* /QF | CAATGAGCCTATGGGAGTGG | qRT-PCR |
| *sclR* /QR | ATCTTCGCCCGAGTGGTT |
| *aflR*/QF | AAAGCACCCTGTCTTCCCTAAC | qRT-PCR |
| *aflR*/QR | GAAGAGGTGGGTCAGTGTTTGTAG |
| *aflS*/QF | CGAGTCGCTCAGGCGCTCAA | qRT-PCR |
| *aflS*/QR | GCTCAGACTGACCGCCGCTC |
| *aflC*/QF | GTGGTGGTTGCCAATGCG | qRT-PCR |
| *aflC*/QR | CTGAAACAGTAGGACGGGAGC |
| *aflD*/QF | GTGGTGGTTGCCAATGCG | qRT-PCR |
| *aflD*/QR | CTGAAACAGTAGGACGGGAGC |
| *aflK*/QF | GAGCGACAGGAGTAACCGTAAG | qRT-PCR |
| *aflK*/QR | CCGATTCCAGACACCATTAGCA |
| *aflO*/QF | GATTGGGATGTGGTCATGCGATT | qRT-PCR |
| *aflO*/QR | GCCTGGGTCCGAAGAATGC |
| *aflP*/QF | ACGAAGCCACTGGTAGAGGAGATG | qRT-PCR |
| *aflP*/QR | GTGAATGACGGCAGGCAGGT |
| *aflQ*/QF | GTCGCATATGCCCCGGTCGG | qRT-PCR |
| *aflQ*/QR | GGCAACCAGTCGGGTTCCGG |

**Supplemental figures**


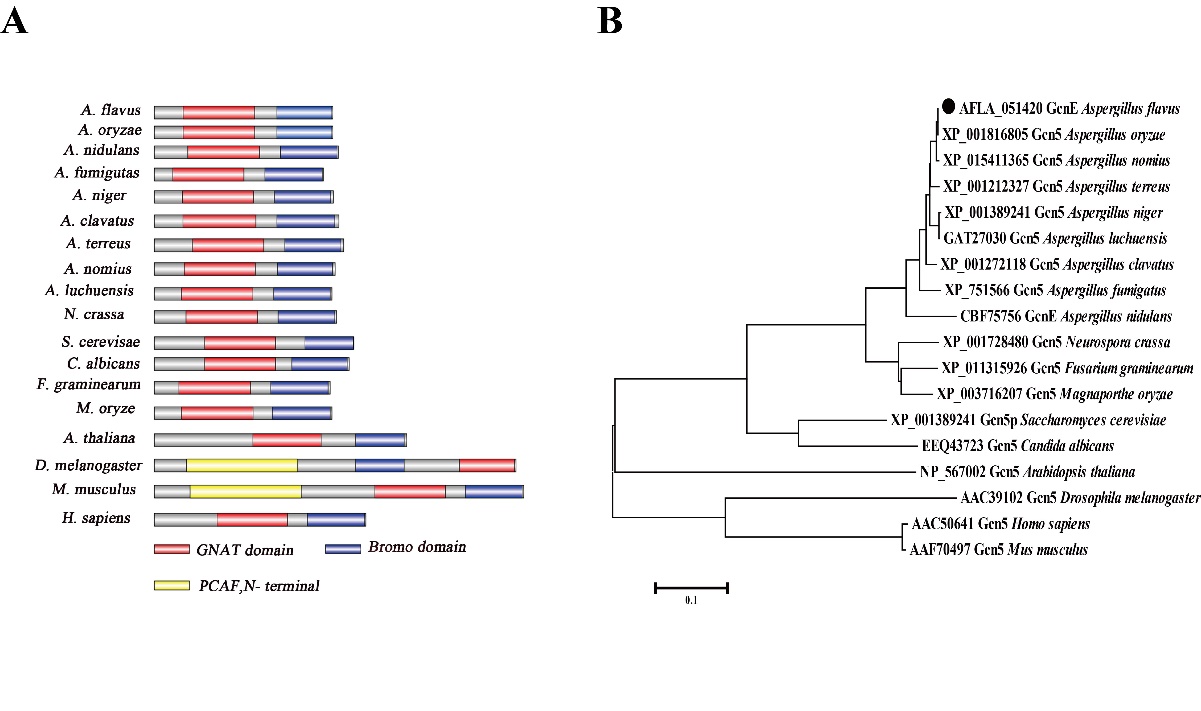


**Figure S1**


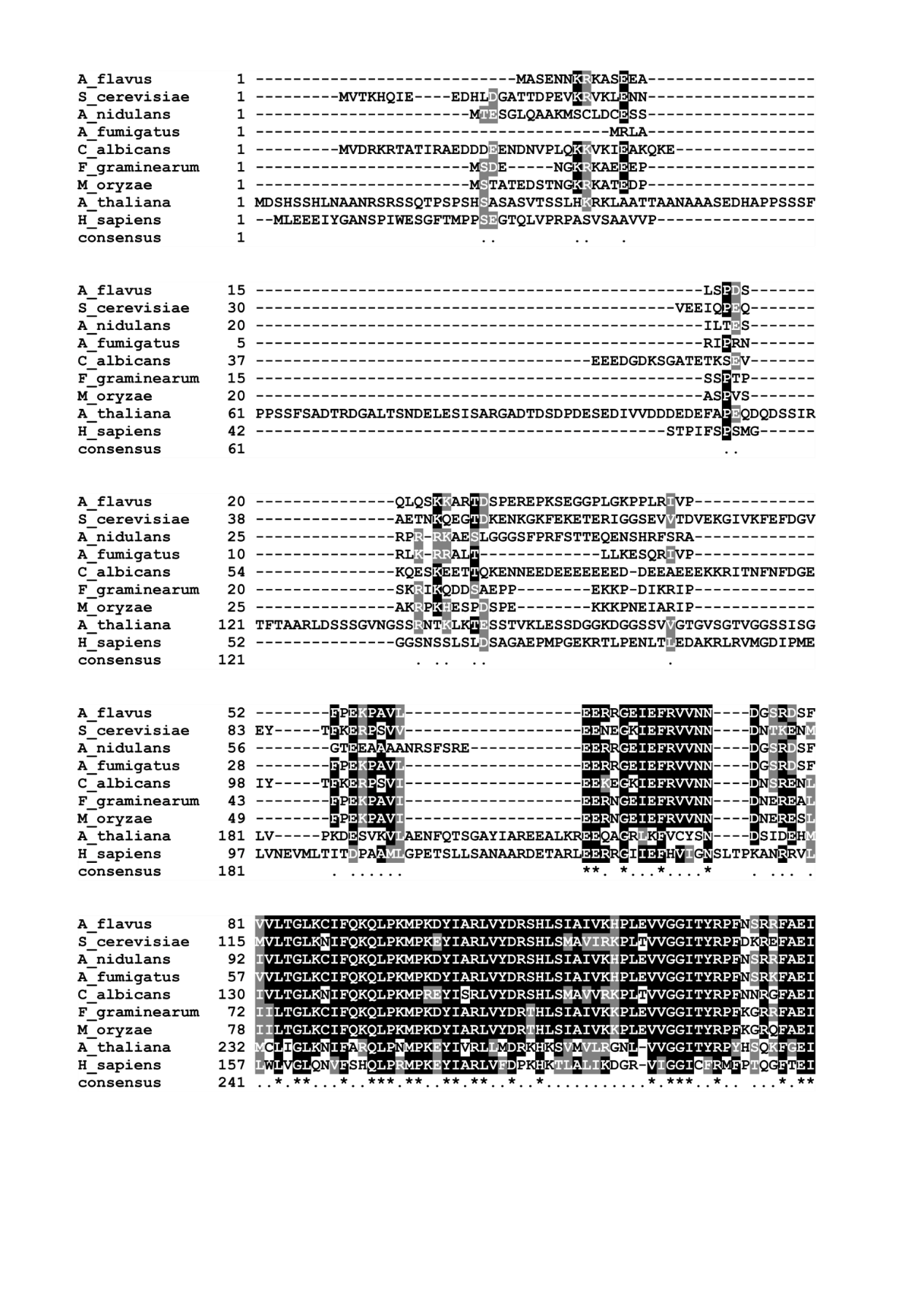

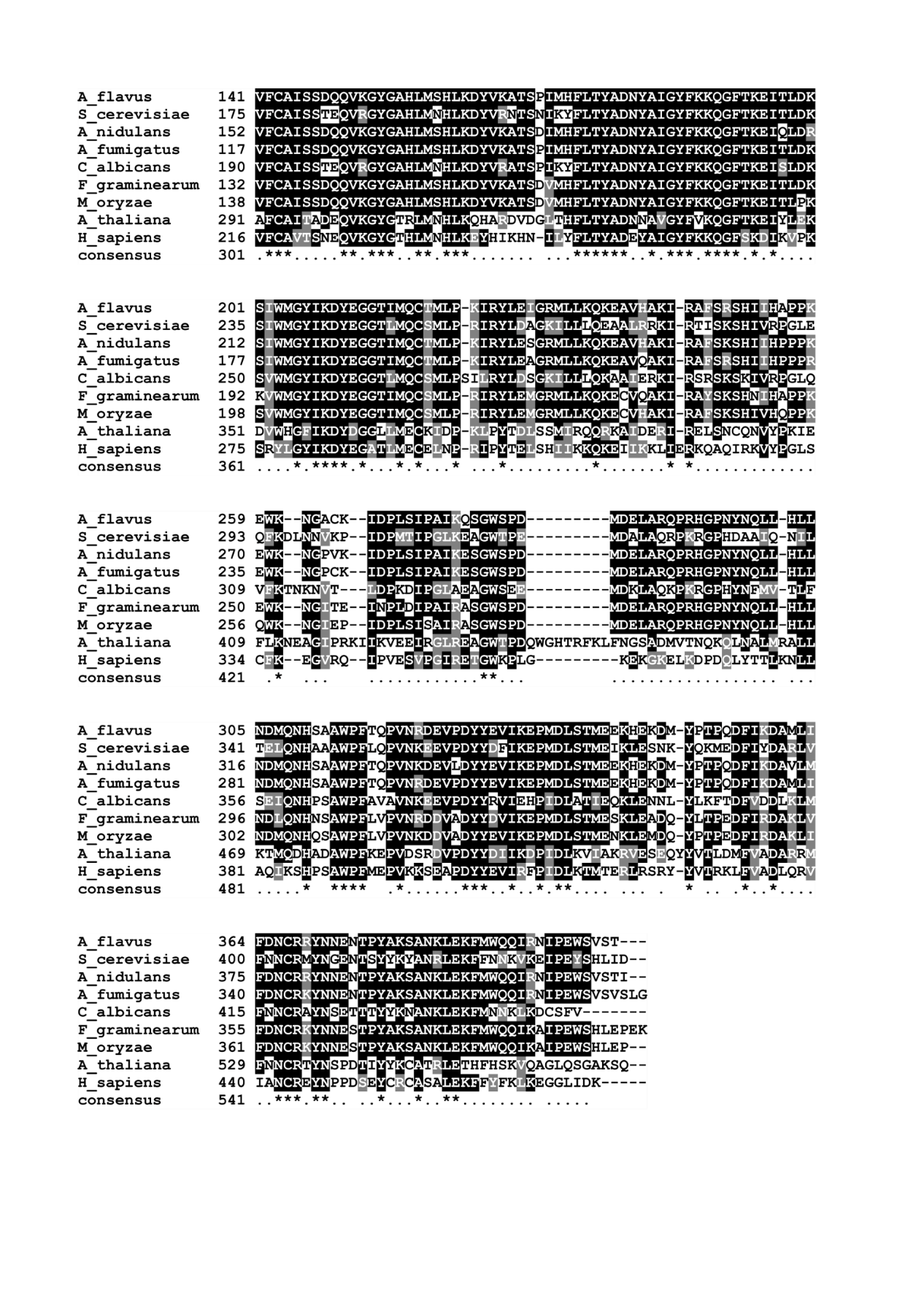


**Figure. S2**


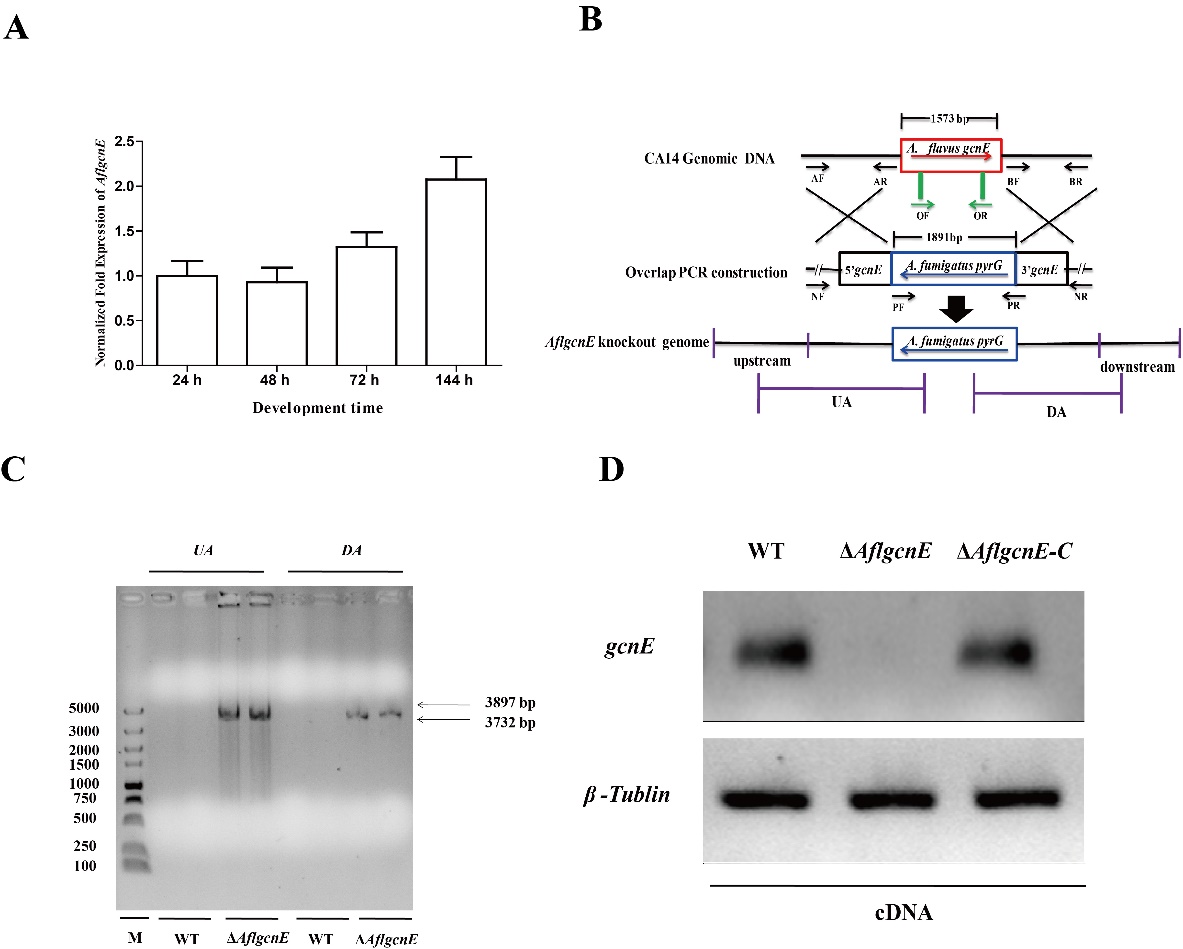


**Figure. S3**


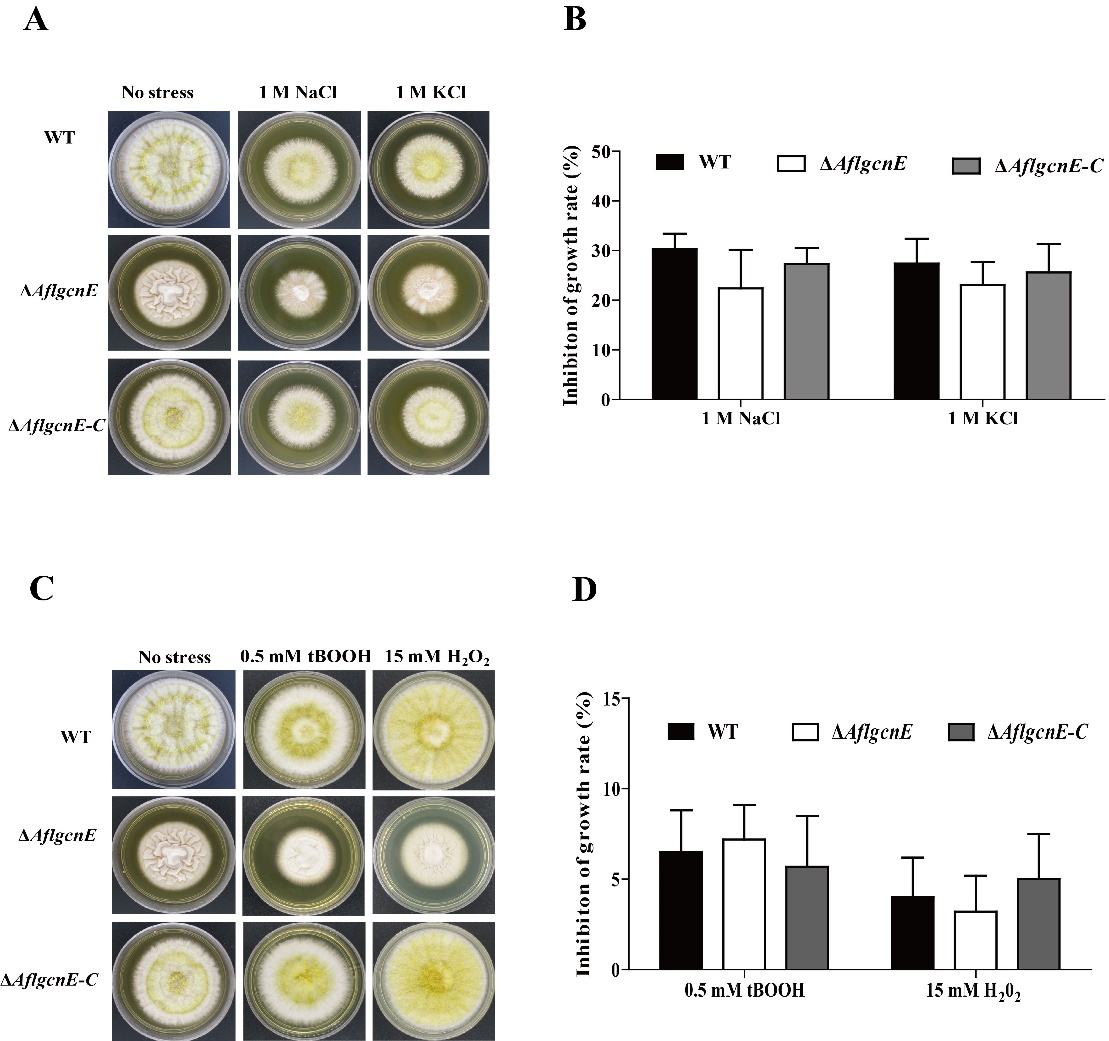


**Figure. S4**
